# Septic Mesenteric Venous Thrombophlebitis: A Rare Complication of Acute Appendicitis

**DOI:** 10.1155/2011/858563

**Published:** 2011-11-20

**Authors:** Stylianos Kykalos, Georgios C. Sotiropoulos, Spiridon Vernadakis

**Affiliations:** Department of General Visceral and Transplantation Surgery, University Hospital Essen, Hufelandstr. 55, 45147 Essen, Germany

## Abstract

Mesenteric venous thrombophlebitis represents a very rare complication of acute appendicitis. Based on the findings of a 45-year-old patient with mesenteric venous thrombophlebitis due to acute appendicitis, we herein describe the diagnostic difficulties and therapeutic options in this uncommon disease. The treatment in our case consisted of simple appendectomy and perioperative anticoagulation therapy.

## 1. Introduction

Mesenteric vein thrombophlebitis may be induced by hypercoagulable, malignant, or septic conditions. Although its incidence due to septic conditions is nowadays pretty low, the related mortality, especially by late diagnosis, is higher as 30% [1]. Abdominal pain, which may worsen after food intake and over time, diarrhea, fever, or gastrointestinal bleeding are some typical but not specific symptoms. We report herein a rare case of septic mesenteric venous thrombophlebitis secondary to acute appendicitis.

## 2. Case Report

**Figure 1:**
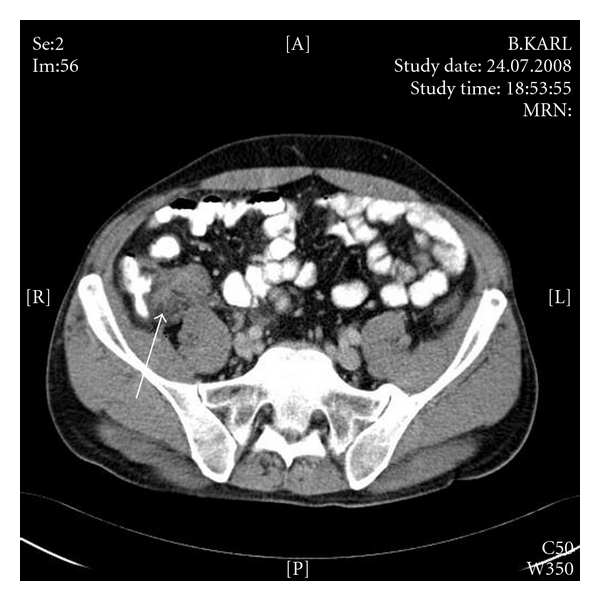
Inflammation of the appendix.

**Figure 2:**
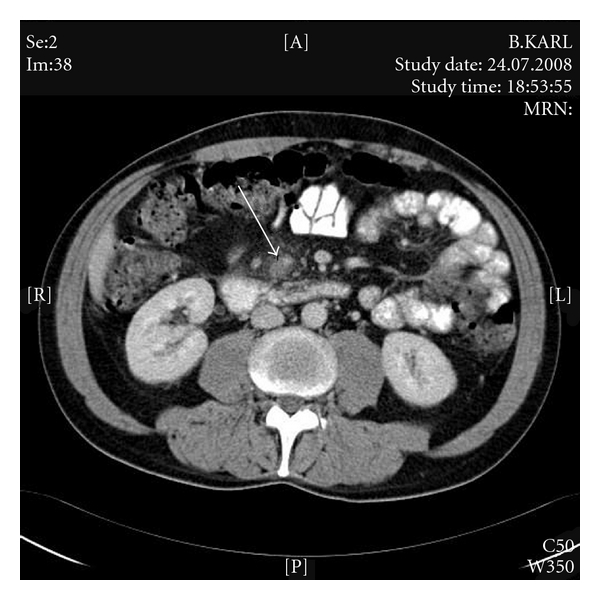
Thrombotic material into the superior mesenteric vein.

A 45-year-old man was transferred in our emergency department due to abdominal discomfort. He mentioned the presence of diffuse abdominal pain since four days with concomitant fever up to 38,5°C and diarrhea. No regular medication intake was mentioned. The clinical examination revealed epigastric pain and the absence of rebound tenderness. Biochemical and hematologist tests (electrolytes, liver and kidney function tests) were within normal laboratory range, with the exception of elevated white blood cells count (WBC = 14.000/mm^3^). Abdominal computed tomography (CT) revealed the presence of an acute appendicitis with acute mesenteric vein thrombosis (Figures 1 and 2). Particularly, CT scan of the pelvic region (Figure 1) showed inflammation of the appendix (arrow) with marked periappendiceal infiltrates, while few slices above a thrombus into the superior mesenteric vein (arrow) were detected (Figure 2), without the presence of ascites or splenomegaly. Apparently, the septic allocation passed through the ileocolic vessel to the superior mesenteric vein, predisposing its thrombosis.

With these findings and prior to operation, the volume flow rate of blood in the portal/superior mesenteric vein was assessed using a duplex ultrasound system.

The patient was treated with emergent open appendectomy and received broad spectrum antibiotics (cefuroxime plus metronidazole). A low-molecular-weight heparin was perioperatively administered, while the postoperative course was uneventful.

The followup included a monthly performed duplex ultrasound, which never revealed thrombus expansion into portal or splenic vein. Furthermore, no splenomegaly or cavernosus transformation of the portal vein was observed. A remarkable thrombus shrinkage was detected during his last examination. The patient remained asymptomatic during the six-month follow-up period.

## 3. Discussion

Every septic condition that drains into the portal system can lead to a mesenteric thrombophlebitis or pylephlebitis, which is defined as thrombophlebitis of the portal vein or its tributary veins. Beside appendicitis, colonic diverticulitis, inflammatory bowel diseases, bowel perforation, pancreatitis or cholangitis are potential triggers [1]. Furthermore, malignancies or hypercoagulable conditions may act as predisposing factors [2].

High index of clinical suspicion and imaging play a crucial role in the diagnosis of mesenteric venous thrombophlebitis, as the condition is usually misdiagnosed intraoperatively. CT scan is the most reliable diagnostic tool [3], being able to indicate the presence and extent of thrombus, as well as the primary source of infection. The suggestive findings of this condition are air bubbles and thrombi in the mesenteric and portal venous system. Doppler ultrasound examination is also highly sensitive in confirming portal vein thrombosis and is mostly useful in the followup of these cases.

The current treatment approach is based on aggressive broad-spectrum antibiotic administration (blood cultures are strongly indicated), with immediate surgical removal or drainage of the septic focus (in the present case appendectomy) and anticoagulation therapy. Anticoagulants are administered in an effort to recanalise the thrombotic vein and reduce the possibility of septic embolisation and abscess formation in the liver [2]. In cases where bowel ischemia encounters as complication, a thrombectomy could be indicated.

Concluding, mesenteric vein thrombophlebitis is a rare clinical entity, which may have high morbidity and mortality, especially by misdiagnosis. In most of the cases, as in our report, the conservative approach is actually adequate, given the successful drainage or surgical removal of the septic focus.
